# An In-Vitro Study of the Root Canal Configuration of Mandibular First Premolars in a Bengali Subpopulation Using Cone-Beam Computed Tomography

**DOI:** 10.7759/cureus.75187

**Published:** 2024-12-05

**Authors:** Shamik Datta, Sayantan Mukherjee, Paromita Mazumdar, Shaon Mukherjee

**Affiliations:** 1 Conservative Dentistry and Endodontics, Guru Nanak Institute of Dental Sciences and Research, Kolkata, IND; 2 Conservative Dentistry and Endodontics, Evershine Dental Clinic, Kolkata, IND; 3 Oral and Maxillofacial Radiology, Oromax Imaging, Kolkata, IND

**Keywords:** apical foramen, canal orifice, cone-beam computed tomography, c-shaped canals, lateral canal, mandibular first premolar, root canal morphology

## Abstract

Aim: This study aims to analyze the root canal configuration system of mandibular first premolars in the Bengali subpopulation using cone-beam computed tomography (CBCT).

Materials and methods: Based on Vertucci's classification, the root canal morphology of 100 randomly selected mandibular first premolars in 56 males and 44 females from the Bengali subpopulation of West Bengal was assessed in vitro. The location of the apical foramen, lateral canal, C-shaped canal, types of canal orifice cross-sections, minor constriction diameter, tooth length, and root length were noted. A chi-square test was conducted to test the categorical variables at a 5% significance level.

Results: Vertucci’s type I (75%, n=75), followed by Vertucci’s type V (23%, n=23), were significantly more common in the present study sample. Most of the two-rooted teeth showed a division at the middle one-third (82.8%, n=18). Cross-sectional evaluation of the samples inferred that an ovular cross-section was most common (78%, n=78). All the above-mentioned proportions were significantly higher (P<0.0001). A C-shaped canal was found in 19% (n=19) of the study samples, and significant associations were found.

Conclusion: Vertucci type I and type V canal configurations are the most frequently observed in mandibular first premolars among a Bengali sub-population.

## Introduction

Endodontic treatment failure often results from improper root canal obturation after poor preparation [1]. Mandibular first premolars are challenging due to high flare-up rates and morphological anomalies [2]. Various studies use methods like radiography and micro-CT to examine their morphology.

Advancements in non-destructive three-dimensional imaging, like cone-beam computed tomography (CBCT) and micro-computed tomography (μ-CT), as well as the use of magnification, have increased the number of reports on complex root canal anatomy [1].

Familiarity with tooth anatomy variations across racial groups is crucial. Studies show differing trends in root and canal numbers. Worldwide, 97.21% of premolars have one root, but only 73.55% have one canal, with 23.55% having two canals. Two roots are more common in African-American (16.2%) and Kuwaiti (15%) populations [2]. About 50% of the Indian population reportedly has two canals [3].

The present study was carried out on a group of the Bengali population due to the existent anatomic variations of the Bengali population as an ethnolinguistic group [4]. Furthermore, to the best of our knowledge, literature is sparse regarding the study of anatomical variations in mandibular first bicuspids in a group of the Bengali population. Furthermore, studies have determined that CBCT is a reasonable substitute for µ-CT, with both imaging modalities showing similar accuracy.

This study aims to analyze the root canal configuration system of mandibular first premolars in the Bengali subpopulation based on Vertucci’s classification due to its wide usage and ease of communication [5].

## Materials and methods

Ethical approval was obtained from the Institutional Ethics Committee of Guru Nanak Institute of Dental Sciences and Research (reference no: GNIDSR/IC/21-24/39). The sample size calculation for this study was performed using an estimated proportion (p) of Vertucci's type I, based on a previous study [6], using the formula: $n = \frac{(Z_{\alpha/2})^2 \cdot p \cdot (1-p)}{e^2}$ where n represents sample size, Z_α/2_ is the standardized normal deviation at 95% confidence level (two-tailed), i.e., 1.96, e is the absolute level of precision set at 10%, and p=0.785 is the expected population proportion. Substituting these values into the formula: $n = \frac{(1.96)^2 \cdot 0.785 \cdot (1-0.785)}{(0.1)^2}$ A sample size of 64 teeth was obtained. Thus, the current study required a total sample size of 64 subjects. However, 100 samples were screened.

The study, following the Population, Exposure, Comparator, Outcomes (PECO) format, focuses on a Bengali sub-population (P), evaluates using CBCT (E), compares across age and gender (C), and investigates various aspects of root canal anatomy, including configuration across coronal, middle, and apical thirds; presence of lateral canals and inter-canal communications; shapes of canal orifices (round, oval, ribbon-shaped); position of the apical foramen; presence of C-shaped canals; diameter of the minor constriction; tooth length; and root length (O).

Sample collection based on ethnic groups

Adequate consideration of ancestral history was crucial in selecting participants for this study. Criteria included verification that both parents and grandparents spoke Bengali as their mother tongue, with no history of marriage outside the Bengali community or ancestral origins from Bangladesh, despite residing in West Bengal. The study utilized freshly extracted mandibular first premolars from Bengali individuals aged 15-40 years, scheduled for extraction due to reasons unrelated to this study. Teeth meeting criteria (e.g., absence of visible fractures, open apices, deformities) were included, while those with crowns, prior endodontic treatment, severe caries, root resorption, calcification, or fractures identified in radiologic and CBCT exams were excluded.

Analysis and expertise

Two calibrated endodontists independently evaluated CBCT scans using a Carestream CS 9300 machine (Carestream Health, Inc., USA), configured with a 5 cm × 5 cm field of view, 90 µ voxel size, 80 kvp, 8 ma, and 20 seconds exposure. In cases of discrepancy, an oral radiologist was consulted for consensus.

Simultaneously, the assessment included recording the division of the canal into coronal, middle, and apical thirds; identification of lateral canals and inter-canal communications; determination of canal orifice shapes (round, oval, or ribbon-shaped); localization of the apical foramen; identification of C-shaped canals; measurement of minor constriction diameter, tooth length, and root length.

Statistical analysis

The collected data were tabulated in Microsoft Excel 2021 (Microsoft Corporation, Redmond, USA) and analyzed using IBM SPSS Statistics for Windows, Version 26 (Released 2019; IBM Corp., Armonk, New York, United States). Categorical variables, including root canal configurations and types of canal orifices, were assessed for inter-rater agreement using Cohen's kappa statistic. Quantitative variables such as the size of the apical foramen, root length, and tooth length were analyzed for intra-rater reliability using the intraclass correlation coefficient (ICC). The statistical tests included chi-square tests for categorical variables and one-way analysis of variance (ANOVA) between age groups, supplemented by independent samples t-tests between genders post-normality assessment. The P-value of ≤0.05 was considered as the level of significance.

## Results

Inter-rater reliability was found to be excellent, with Cohen's kappa coefficient yielding a value of 0.985, indicating strong agreement for categorical variables. Similarly, ICC analysis for quantitative variables showed high reliability with a coefficient of 0.99.

Out of the 100 mandibular premolars, the majority of the study participants belonged to the age group of 21-30 years (60%, n=60) and were males (56%, n=56) (Table 1).

**Table 1:** Morphological characteristics of the mandibular first bicuspid in the present study population a: analyzed by Pearson’s chi-square test of proportions; *: statistically significant (P<0.05); **: highly statistically significant (P<0.01)

Morphologic characteristics			Frequency (%)	95% confidence intervals	χ^2 ^value	P-value^a^
Number of roots	1		99 (99%)	94.55-99.95	96	<0.001**
	2		1 (1%)	0.05-5.45		
Number of canals	1		75 (75%)	65.7-82.45	27	<0.001**
	2		25 (25%)	17.55-34.3		
Vertucci’s type	I		75 (75%)	66.76-83.3	204	<0.0001**
	II		1 (1%)	0.05-5.45		
	III		1 (1%)	0.05-5.45		
	V		23 (22%)	15.001-31.07		
Division/unification		Levels				
	Division	Apical 3rd	4 (18.2%)	7.3-38.5	11.25	<0.0001**
		Middle 3rd	18 (82.8%)	58.1-90.34		
	Unification	Middle 3rd	1			
Location of apical foramen	Central		17 (17%)	10.89-25.55	43.6	<0.0001**
	Lateral		83 (83%)	74.45-89.11		
Number of Lateral canals	1		20 (83.3%)	64.15-93.32	10.7	0.001**
	2		4 (16.7%)	6.68-35.85		
Position of lateral canals	Apical		23 (95.83%)	79.76-99.79	20.2	0.001**
	Middle		1 (4.17%)	0.21-20.24		
Cross-section of canal orifice	Oval		78 (78%)	68.93-85	91.9	<0.0001**
	Ribbon		17 (17%)	10.89-25.55		
	Round		5 (5%)	2.15-11.18		

**Figure 1:**
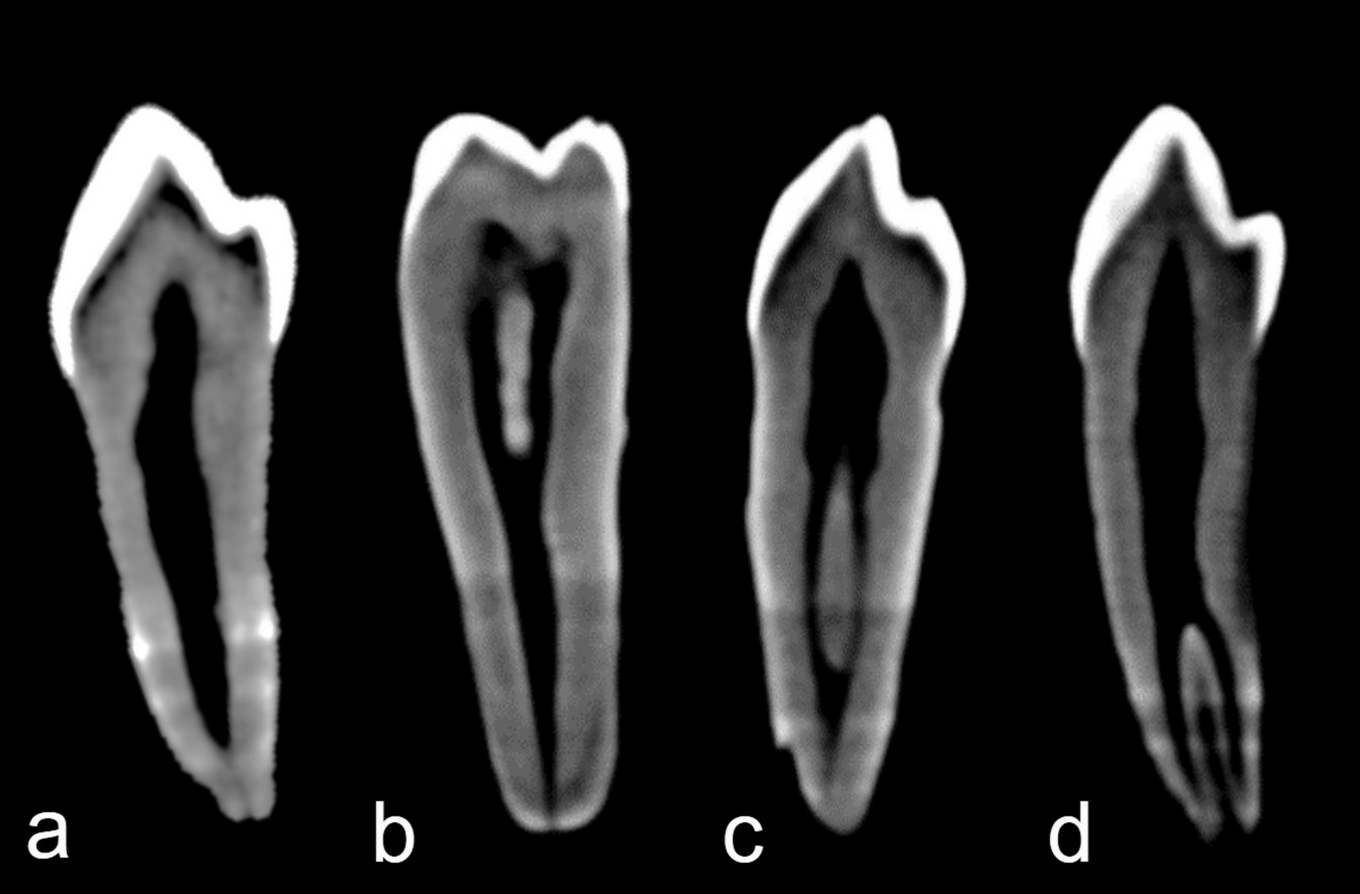
Cone beam computed tomographic images (sagittal section) of the types of canal configurations according to Vertucci’s classification found in the present study a) Type I, b) Type II, c) Type III, and, d) Type V

Table 1 summarizes the morphologic characteristics of the mandibular premolars in the present study population. It was observed that Vertucci’s type I (75%, n=75), followed by Vertucci’s type V (23%, n=23) (Figure 1), were the most commonly found canal morphology in the present study sample (P<0.0001), and hence the presence of a single canal was the most prevalent (75%, n=75). Most of the two-rooted teeth showed a division at the middle one-third (82.8%, n=18). A laterally placed apical foramen was most frequent (83%, n=18). A single lateral canal was most commonly found (83.3%, n=20), presently in the apical third region (95.83%, n=23). Cross-sectional evaluation of the samples inferred that an ovular cross-section was most common (78%, n=78). All the proportions mentioned above were significantly higher (P<0.0001).

**Figure 2:**
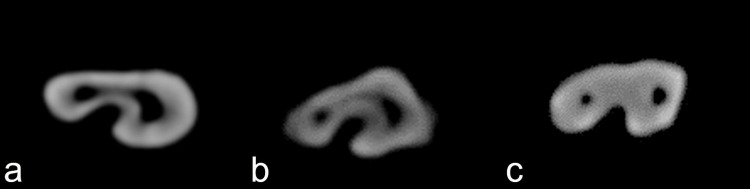
Cone beam computed tomographic images (axial section) of the types of C-shaped canal configurations according to fan’s classification found in the present study a) Type C1, b) Type C2, and, c) Type C3

C-shaped canal was found in 19% (n=19) of the study samples (Figure 2).

Fan’s Type C3 was the most predominantly (82.76%, n=24) found C-shaped type at all levels of cross-section and it was significantly higher than that of C2 (10.34%, n=3) and C1 (6.89%, n=2). When stratified by cross-sections, no C-shaped canal was prevalent in the coronal third (Table 2).

**Table 2:** Morphological characteristics of the C-shaped canals NS: not statistically significant (P>0.05)

Types and levels	Coronal 3rd	Middle 3rd	Apical 3rd	Total	χ^2 ^value	P-value
C1	0 (0%)	1 (9.1%)	1 (5.55%)	2 (6.89%)	1.391	0.49 (NS)
C2	0 (0%)	2 (18.2%)	1 (5.55%)	3 (10.34%)		
C3	0 (0%)	8 (72.73%)	16 (88.89%)	24 (82.76%)		

However, the distribution of the canal types according to the cross-section levels was not significant (P>0.05). The mean diameter of the minor constriction was found to be 0.186±0.06 mm. All the variables were cross-tabulated with age and gender; however, no significant association was found (P>0.05).

## Discussion

Understanding tooth anatomy is crucial for endodontic treatment, as improper root canal obturation leads to failure. Typically, roots have a single canal, but complexities with multiple exits are common [7].

Most of the studies conducted previously on mandibular bicuspids were carried out mostly on the Tamil population [8,9] and the Gujarati population [10,11], but the teeth samples used in the current study mainly belonged to the Bengali populace, which has not been studied expansively.

In the present study, only 1% (n=1) were found to have two roots (one tooth), which is similar to the findings of Velmurugan et al. [8] and Jain and Bahuguna [11]. However, Parekh et al. [10] reported a similar prevalence of single roots and canals and two roots and canals, which is 50% each.

The tooth length recorded in this study for the first mandibular premolar ranged from 17.1 to 26.2 mm, and the average length was 21.1 mm. The finding is nearly similar to the average length of 21.2 mm with a range of 17-26 mm that was recorded for an Indian population by Jain et al. [11]. The diameter of the minor constriction was found to be 0.18±0.06 mm with a range from 0.1 to 0.3 mm. However, Keleş and Keskin (2020) [12] reported an average minor constriction diameter of 0.25 mm.

In the present study, most of the study samples possessed a single root (99 teeth). Seventy-five of the study samples had a single canal. The above-mentioned findings corroborate the findings of the study by Cleghorn et al. [13] and Singh and Pawar (2014) [14], who, in their studies, respectively, reported 97% and 94% of the first premolars to have a single root, and 76% of them had a single canal.

In the current study, the second most prevalent root canal configuration was found to be Vertucci’s type V (23%, n=23). Similar findings were noted in the study of Jain et al. [11] and Shrestha et al. [15]. Where they found type V canal configuration in 17.4% and 18.6%, respectively, of their study population and recorded it to be the second most commonly found root canal configuration. However, Parekh et al. [10] and John et al. [6] reported type IV root canal configuration to be the most dominant, while Sandhya et al. [9] and Karobari et al. [16] reported type II canal configuration to be the most predominant, only second to type I root canal configuration. The reason for the variation in the root canal morphology can be attributed to the different populations on which the studies were carried out. Age seems to influence canal morphology; as age increases and secondary dentine continues to deposit, it can alter the structure of preformed root canals. Incomplete root formation in a population below 15 years can alter the canal morphology [12]. However, in the present research, no influence of age or gender was found on the root canal morphology (P>0.05). However, Karobari et al. [16] reported an association between age and canal variations. The reason can be attributed to a different ethnic group that was assessed in the present study, and also a lesser frequency of samples was retrieved from the age group of 15-20 years.

A single apical foramen has been reported most commonly in previous studies as reported by Kottoor et al. [3] in their systematic review. Similar results were evident in the current study where the majority of the foramen opened laterally.

Alkaabi et al. [17] found in their study that the apical foramen opened more laterally than centrally, which was accordant with the results of the present study.

Accessory canals are another common feature in the mandibular first premolars. Accessory canals were reported in 24 teeth in the present study, where a single lateral canal was found in 20 teeth, whereas the rest of the four teeth showed two lateral canal openings. Also, most of the lateral canals were observed in the apical region (23 teeth). The findings of the present study go closely to the study of Kartal and Yanikoğlu [5], who reported 23.49% having multiple canal openings at the apex. However, Cleghorn et al. [13] found the incidence of lateral canals in only 8.2% of the teeth.

Concerning the shape of the canal orifice, the majority of the canal orifices were found to be round (78%, n=78), while only 17% were found to be ribbon in shape, and the rest were oval (5%). The results are somewhat in contrast to the study by Jain et al. [11], who reported a predominant existence of an oval-shaped canal (52.17%) in the study. This discrepancy can be ascribed to the different ethnicities of the population from which the samples were obtained.

Mandibular first premolar architecture varies, with a considerable proportion of C-shaped canal configurations [3]. In the present study, the prevalence of C-shaped canal configuration was found to be 19% (n=19), which corroborated the findings of previous studies where a prevalence of 19.48% was found [18]. Astonishingly, the results are in contrast to the findings in Indian studies where the prevalence of C-shaped canal prevalence has been found in the range of 1-10.7% [8,9,11,19]. The most commonly found cross-section of a C-shaped canal was the C3 type (82.76%). The proportion of C-shaped canals located in the apical region was found to be the highest (66.67%), followed by the middle third (33.33%). However, no C-shaped configuration of the canal was found in the coronal region. The findings of the present study go closely to the study by Zhang et al. [20].

Nevertheless, the study had a few potential limitations. Inherent distortions associated with CBCT scans may have been present. Therefore, it should be borne in mind that the latter may be inconclusive in the identification of some varieties of root canal morphologies [21,22] and is not advised to be taken routinely in all non-surgical endodontic treatment cases [23]. Also, internal reliability could not be assessed due to a single examiner evaluation, leading to errors due to personal interpretation.

## Conclusions

The present study on the morphological characteristics of mandibular first premolars in the Bengali subpopulation reveals notable findings. The majority of these premolars exhibited a single root (99%) and a single canal (75%), with Vertucci's type I canal configuration prevailing at 75%. Apical division predominantly occurred in the middle third of the root (82.8%), with the apical foramen typically located laterally (83%). Lateral canals were found in 20% of cases, primarily in the apical third (95.83%), and oval-shaped canal orifices were predominant (78%). In contrast, C-shaped canal configurations were less common, with type C3 being the most prevalent (82.76%), predominantly observed in the middle and apical thirds of the root. These detailed morphological insights underscore the variability of root canal anatomy within the Bengali population, emphasizing the need for personalized treatment approaches in endodontic practice.

Further studies with larger samples, lower voxel size in vivo, or μ-CT are needed to better understand mandibular first premolar morphology variations by age, gender, and side.
